# Tracing Hidden Herbivores: Time-Resolved Non-Invasive Analysis of Belowground Volatiles by Proton-Transfer-Reaction Mass Spectrometry (PTR-MS)

**DOI:** 10.1007/s10886-012-0129-3

**Published:** 2012-05-18

**Authors:** Holger Danner, Devasena Samudrala, Simona M. Cristescu, Nicole M. Van Dam

**Affiliations:** 1 Department of Ecogenomics, Institute for Water and Wetland Research (IWWR), Radboud University, PO Box 9010, 6500 GL Nijmegen, The Netherlands; 2 Life Science Trace Gas Facility, Institute for Molecules and Materials, Radboud University, Nijmegen, The Netherlands

**Keywords:** Chemical ecology, Root herbivory, Trace gas analysis, Induced indirect defense, Mass spectrometry, Volatile organic compound (VOC)

## Abstract

Root herbivores are notoriously difficult to study, as they feed hidden in the soil. However, root herbivores may be traced by analyzing specific volatile organic compounds (VOCs) that are produced by damaged roots. These VOCs not only support parasitoids in the localization of their host, but also may help scientists study belowground plant-herbivore interactions. Herbivore-induced VOCs are usually analyzed by gas-chromatography mass spectrometry (GC-MS), but with this off-line method, the gases of interest need to be preconcentrated, and destructive sampling is required to assess the level of damage to the roots. In contrast to this, proton-transfer-reaction mass spectrometry (PTR-MS) is a very sensitive on-line, non-invasive method. PTR-MS already has been successfully applied to analyze VOCs produced by aboveground (infested) plant parts. In this review, we provide a brief overview of PTR-MS and illustrate how this technology can be applied to detect specific root-herbivore induced VOCs from *Brassica* plants. We also specify the advantages and disadvantages of PTR-MS analyses and new technological developments to overcome their limitations.

## Introduction

Belowground herbivores can cause substantial damage to plant roots, which in many cases has a more severe impact on plant fitness than shoot damage (Gerber et al., 2007; Johnson et al., 2007). Nevertheless, interactions between belowground herbivores and their hosts have been much less-studied than those of their aboveground counterparts. One of the reasons is the obscurity of root-herbivore interactions in the soil, which also means that root damage cannot be assessed as easily as shoot damage. Plants generally need to be sampled destructively to assess how much and where root feeding has occurred. To overcome this drawback and to non-invasively visualize the activities of root herbivores, techniques such as X-ray tomography and magnetic resonance imaging (MRI) have been successfully applied (Johnson et al., 2007; Jahnke et al., 2009). In addition, the development of new methods, for example, root area determination by using electrical potential measurements, may lead to novel approaches that help to monitor the feeding activities of root herbivores *in vivo* (Cao et al., 2010). Here, we present a novel approach to tracing the feeding activities of root herbivores that involves the detection of herbivore-induced volatile organic compounds (VOCs) as a method for damage assessment (van Tol et al., 2001; Rasmann et al., 2005; Kaplan et al., 2008).

Despite the paucity of data, it has become evident that responses induced below ground in many respects resemble those found in aboveground plant-herbivore interactions. Just as in shoots, responses induced by herbivores may be both local and systemic, either within the root system or the whole plant, and comprise a wide range of defense compounds, such as alkaloids, phenolics, cardiac glycosides, and glucosinolates (Kaplan et al., 2008; Rasmann et al., 2009; van Dam, 2009; Hiltpold et al., 2011; Pierre et al., 2011a). In addition, root herbivory leads to the induction of volatile organic compounds (VOCs) that can be involved in indirect defenses below ground, by attracting the enemies of the attackers (van Tol et al., 2001; Rasmann et al., 2005; Pierre et al., 2011b). The role of VOCs might be even more important for belowground communities in the rhizosphere, as they serve herbivores and parasoitoids as cues for host localization in an environment where visual cues are lacking (Rasmann et al., 2005; van Dam, 2009). Indeed, recent studies have revealed various root-produced VOCs that play a role in plant-environment interactions. Maize roots attacked by larvae of the Western cornworm (*Diabrotica virgifera virgifera*) emit (*E*)-β-caryophyllene, a sesquiterpenoid that attracts entomopathogenic nematodes (Rasmann et al., 2005), while *Brassica* plants infested with the larvae of the cabbage root fly (*Delia radicum*) emit sulfides that attract ground-dwelling predatory beetles (Ferry et al., 2007) and also various other VOCs that may be important cues for parasitoids of these root herbivores (Neveu et al., 2002; Pierre et al., 2011b).

Many compound classes that have been identified to play a role in belowground plant-environment interactions also are known from aboveground organs. Despite the overlap in defense strategies and compounds, there are striking differences in the VOCs produced by roots and shoots. For example, green leaf volatiles (GLVs) are commonly emitted by aboveground tissues of almost all higher plants after damage (Hansson et al., 1999; Barth and Schmid, 2001). However, they are not emitted when plant roots are artificially damaged or infested by herbivores (Steeghs et al., 2004), although they can be detected in minute amounts when plant roots are ground up (Matthias Erb, pers. comm.). Furthermore, the emission of sulfides, which often decreases when *Brassica* plants are damaged by aboveground herbivores (Blaakmeer et al., 1994; Geervliet et al., 1997), is strongly enhanced in roots of belowground-infested *Brassica* plants (Blaakmeer et al., 1994; Geervliet et al., 1997; Ferry et al., 2007; Soler et al., 2007). This suggests that root volatile “bouquets” may have a different composition from shoot VOC profiles. These differences may be related to differences in the performance of these compounds in soil environments. Properties such as polarity, boiling point, and solubility determine the degradation, adsorption to soil particles, and the distance over which a compound can disperse through soils, which in turn are important factors for the perception by soil biota. At present, the diversity of herbivore-induced VOCs released by aboveground plant organs appears to be greater than that in roots. It must be noted, however, that there still is a paucity of data on root specific VOCs, which leads to a bias, underestimating VOCs from roots.

In addition to local VOC responses, root herbivores also may induce systemic responses in shoots. The activities of root herbivores not only affect aboveground herbivores that are ovipositing and feeding on the leaves of the same plant (Bezemer et al., 2004; Anderson et al., 2011), but also alter the behavior of organisms at higher trophic levels—such as parasitoids and predators—foraging above ground (Rasmann and Turlings, 2007; Soler et al., 2007, this issue). The effect on aboveground higher trophic levels can either be mediated through changes in the host plant quality elicited in root-induced plants, such as proteinase inhibitors and the accumulation of secondary metabolites, or *via* changes in the volatile bouquets of root-induced plants that render these plants less attractive (Rasmann and Turlings, 2007; Soler et al., 2007). Changes in VOC emissions due to root herbivory can be detected in both belowground and aboveground tissues. Such root-induced changes in VOC emissions possibly can be exploited as indicators of root damage by herbivores without harvesting the plant. In order to do so, we need sensitive and non-invasive techniques that are capable of detecting minute changes in VOC emissions. Proton-transfer-reaction mass spectrometry (PTR-MS) is an on-line technique that allows the sensitive assessment of plant VOCs in real-time. In this review, we discuss the potential, possibilities, and pitfalls of using PTR-MS for the non-invasive and on-line analysis of VOCs induced by root herbivores in comparison to more traditional techniques applied in VOC research.

## VOC Analysis Using GC Platforms

Plants emit substantial amounts of their assimilated carbon as VOCs: Up to 10 % of their carbon assimilation can be released in this way (Peñuelas and Llusià, 2004). These emissions mainly consist of isoprene, a short chain (C5) hydrocarbon. The highly diverse class of higher isoprenoids (>30,000 different structures are described to date; Connolly and Hill, 1991) contributes smaller proportions, and their emission rates are often correlated with biotic and abiotic stressors. In the early days of chemical ecology, around three decades ago, the ability to investigate gaseous emissions from plants focused on the major peaks in the chromatogram. With the progress of analytical technologies, we are more and more approaching whole metabolome analyses, which is important, since minor compounds in the background of a complex volatile blend can contribute significantly to the biological activity of that blend (Mumm and Hilker, 2005; van Dam and Poppy, 2006).

The emission rates of plant VOCs usually are very low, ranging from a few nanograms to micrograms per gram plant dry weight, released per hour. At present, reported emission rates are particularly difficult to compare. In the chemical ecology literature, emission values are either presented in relation to plant weight (dry or fresh), or as relative emissions (e.g., Geervliet et al., 1997; Pierre et al., 2011b). This is, most likely, due to the fact that it would require many authentic standards to properly quantify each VOC in GC-MS analyses, and these are often difficult to obtain. Frequently, it also is a problem of units that prevents direct comparisons between published studies, especially between GC-MS and PTR-MS analyses.

A conversion of the unit commonly used for VOC emissions, in ng·g^−1^[plant weight]·h^−1^, into mixing ratios (in parts per billion volume, a common non SI-unit to report PTR-MS results) will give us this opportunity. We can approximately convert emissions by the following formula^1^:

1 $$ {\text{Emissio}}{{\text{n}}_{{ppbv}}} = \frac{{{\text{Emissio}}{{\text{n}}_{{g \cdot {g^{{ - 1}}}\left[ {DW} \right] \cdot {h^{{ - 1}}}}}} * 24.5\frac{\text{l}}{\text{mol}} * {{\text{m}}_{{plant}}}}}{{{{\text{M}}_r} * {{\text{V}}_{{air}}}}} $$

Here, we give an example of this conversion for green leaf volatiles (GLVs), which are one of the most widespread VOC classes in the plant kingdom, at least above ground (Table 1). Because of the molecular weight, which has to be taken into account for the conversion, each compound contributes different ratios to the total emission, depending on the unit the value is described with. The GLV (*Z*)-3-Hexenol, for example, was emitted at a rate of 153.6 ng·g^−1^ [dw]·h^−1^, which is 43 % of all GLVs in the herbivore treatment, but when expressed as a mixing ratio (ppbv), the same compound constitutes 53 % of all GLVs (Table 1). Depending on the context, for example for insect physiology, the values might be biologically more informative when presented on a number-of-molecule basis, whereas in atmospheric chemistry the gram-based units might be preferred.

**Table 1:** Conversion of units commonly found in the literature for plant VOC emissions with an example of gypsy moth-induced green leaf volatiles (GLVs) of poplar (*Populus trichocarpa*), adapted from Danner et al. (2011)

Compound	M_r_^a^	Emission [ng · g^−1^ DW^b^ · h^−1^]		Emission [ppbv^c^]	
	[g · mol^−1^]	Control	Herb.	Control	Herb.
(*Z*)-3-Hexenol	100.2	9.4	153.6	0.16	2.61
(*Z*)-3-Hexenylacetate	142.2	19.1	153	0.22	1.83
Hexyl acetate	144.2	1.1	44.7	0.01	0.53

^a^M_r_—relative molecular weight

^b^DW—dry weight

^c^ppbv—parts per billion volume

Due to low emission rates, the VOCs sampled from the plant headspace usually need to be pre-concentrated on adsorbents before they can be analyzed on gas chromatography (GC) platforms. Most commonly, plant VOCs are sampled on tubes filled with polymer materials, such as Tenax, Porapaq, Carbopack, and charcoal, or on solid phase micro-extraction fibers (SPME; D’Alessandro and Turlings, 2006; Tholl et al., 2006; Birkett, 2010). In view of this necessity to preconcentrate the sample before analysis, the procedure involves collection periods in the range of minutes to hours, which prevents highly time-resolved measurements of VOC emissions. Additionally, the sampling procedure may cause contaminations to be introduced when solvents are used to elute VOCs from the tubes before injection on the GC. With other sampling techniques this can be avoided. Using direct thermodesorption (TD) tubes, the VOCs are thermally desorbed from the packing material and transferred directly to the GC injector port in the gaseous phase (e.g., Pierre et al., 2011b) In both cases, however, the relatively high temperatures, essential for rapid desorption or evaporation of the solvent in the injector port, may cause the VOCs to break-down or to be converted into other components (de Kraker et al., 1998). Certain VOCs, such as sabinene and α-pinene, also degrade to some extent as a result of reactions with the adsorbent surface (Rothweiler et al., 1991; Coeur et al., 1997). Moreover, depending on the packing materials of the sampling tubes, selective breakthrough of certain compounds, such as isoprene and other short-chain hydrocarbons, may occur, which makes the analysis less quantitative for these compounds (Dettmer et al., 2000). By contrast, PTR-MS has the potential to sample VOCs on-line and with high sensitivity (pptv), without the need for pre-concentration, thereby avoiding many of the above-mentioned drawbacks. Additionally, the instrument operates at much lower temperatures (around 50°C) which reduces the formation of chemical artifacts (Hansel et al., 1995).

## Proton-Transfer-Reaction Mass-Spectrometry (PTR-MS)

About 15 years ago, PTR-MS emerged as a powerful tool for monitoring VOCs. Whereas conventional MS technology is often based on electron ionization (EI), which results in extensive fragmentation providing rich ion fragments, PTR-MS relies on chemical ionization (CI), a soft ionization method with few or no ion fragments in the mass spectra. A detailed description of the PTR-MS technology has been published elsewhere (Hansel et al., 1995; de Gouw et al., 2003; Boamfa et al., 2004). Here, we briefly outline the main characteristics of the PTR-MS technology in so far as they are essential to be able to evaluate its opportunities and limitations for plant VOC analyses.

**Fig. 1:**
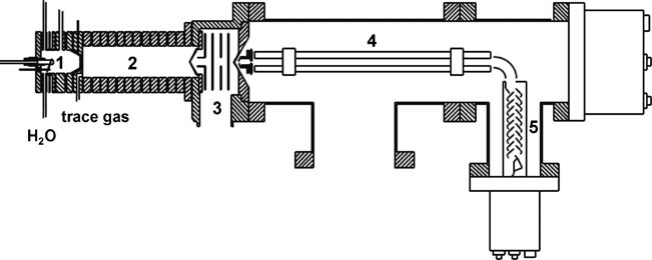
Schematic representation of a typical PTR-MS. The instrument consists of an ion source (1) in which H_3_O^+^ primary ions are produced, a drift tube (2), where the trace gases from samples are ionized by the proton-transfer reaction with H_3_O^+^ ions, a collisional dissociation chamber (3), where cluster molecules dissociate, and the detection unit, where ions are mass filtered with a quadrupole mass filter (4) and quantified by a secondary electron multiplier (5)

In PTR-MS, a neutral molecule is ionized *via* a CI reaction with H_3_O^+^. The ionized molecules typically form a protonated molecular ion [M + H]^+^, in which M is the molecular mass of the parent molecule. Water or a mixture of water and helium is introduced and the H_3_O^+^ ions are produced by a, mostly hollow, cathode discharge in the primary ion source (Fig. 1, no. 1; Boamfa et al., 2004). Thereafter, the H_3_O^+^ ions enter the reaction chamber, the so-called drift tube (Fig. 1, no. 2), where they are driven by a homogenous electric field and will interact with the trace gas mixture that enters directly *via* an inlet at low gas flow rate (~ 0.5 l/h). Typically, only molecules with a proton affinity higher than that of water (>166.5 kcal mol^−1^) will be ionized by proton-transfer-reactions with H_3_O^+^ ions. Organic compounds such as aldehydes, ketones, alcohols, oxygenated aromatic and aliphatic compounds will be readily protonated (Warneke et al., 2003; Hartungen et al., 2004; Wisthaler et al., 2005; de Gouw and Warneke, 2007).

In addition to the normal proton-transfer-reaction, the H_3_O^+^ and [M + H]^+^ ions can cluster with water molecules in the drift tube, complicating the interpretation of mass spectra. Since the proton affinity of the clusters is higher than that of water, the proton-transfer-reaction with a water cluster will be favored. An important part of the instrument that serves to reduce problems of cluster formation is the collision dissociation chamber (Fig. 1, no. 3). In this intermediate chamber, the cluster ions that leave the drift tube dissociate into a neutral moiety and the initial, protonated trace gas molecule [M + H]^+^. Cluster formation can be reduced further by adapting the reaction conditions of the PTR-MS instrument for this purpose (2 mbar pressure and 120–140 Td field strength in the drift tube).

As described above, the proton-transfer results in few or no fragment ions for most trace gas compounds. Despite this general rule, fragment ions are still detected in the mass spectra for certain compounds, increasingly more with higher kinetic energy in the drift tube (Maleknia et al., 2007). Above all, the fragmentation pattern depends on the structure of the molecules. For example, alcohols break down easily and lose a water molecule *via* dehydration, whereas acetaldehyde or acetone is less likely to dissociate (Boamfa et al., 2004). Although there are extensive resources for EI fragmentation patterns [e.g., National Institute of Standards and Technology (NIST, USA) and Wiley (West Sussex, England)], these spectral libraries cannot be applied as a reference to PTR-MS, in view of the dissimilar ionization methods. Consequently, one needs to determine the fragmentation behavior of the VOCs under study either from the literature, or by reference measurements with authentic standards.

Another aspect to be considered is the back diffusion of air from the drift tube into the ion source, which leads to contaminant ions, such as NO^+^ and O_2_^+^ (de Gouw and Warneke, 2007). PTR-MS of plant VOCs requires the amount of these ions to be reduced, because they transfer their charge to most VOCs without adding a proton, which complicates the identification of compounds. However, higher levels of NO^+^ and O_2_^+^ ions may also be beneficial for detecting specific compounds, such as sulfur-containing glucosinolate breakdown products (Crespo, 2012).

### Quantification and Identification of VOCs

In PTR-MS, regular calibration with an authentic gas mixture is a prerequisite for reliable quantification of trace gases, for example, drift tube humidity can vary, which has an impact on the drift tube reactions. A typical example of a calibration gas mixture consists of acetaldehyde, acetone, isoprene, benzene, toluene, xylene, and α-pinene (covering molecular masses from 32 amu to 136 amu), each at a concentration of 1 ppmv (parts per million volume, ±5 %). The calibration factors obtained for the fixed set of compounds in the certified gas mixture can be used to calculate the calibration factors of other compounds, by taking into account their collision rate constants, transmission efficiency factors, and fragmentation ratios. In this way, ion intensities (expressed as normalized counts per second, ncps) can be converted to absolute concentrations as gas mixing ratios (parts per billion volume, ppbv). PTR-MS can operate in two modes, namely the full mass scan and selective ion monitoring (SIM). The first scans the relative abundance of all detectable masses, and should be regarded as a fingerprint of a given trace gas sample (Steeghs et al., 2004). In contrast, the SIM mode is suitable for recording temporal changes in concentrations of specific trace gas molecules, pre-selected by their mass-to-charge ratios.

The major drawback with PTR-MS remains the identification of compounds, which is notoriously difficult, as each detected mass can either be associated with parent molecules, fragments of parent molecules, and water clusters, or a combination of these. Therefore, the identification of compounds measured by PTR-MS is mostly tentative. Nevertheless, if several compounds with the same nominal mass must be considered as possible candidates in a gas mixture, several methods to distinguish between these compounds can be employed. For example, water clusters can be easily distinguished from compounds undergoing the usual proton transfer reaction by varying the field strength in the drift tube (E/N). Association processes with water are quite sensitive to higher collision energies (E/N), thus, if the intensity of a signal decreases with higher E/N, the signal is contributed by a compound associated with one or more water molecules.

In the same way, the abundance of stable isotopes can provide further information about the identity of a compound. The probability of ^13^C incorporation into a molecule rises in a linear fashion with the number of carbon atoms in that molecule. For example, with the natural ^13^C abundance of 1.1 %, a molecule containing 5 carbon atoms, such as isoprene (*M =* 68) has a chance of 5.5 % to contain exactly one ^13^C. With PTR-MS, isoprene is detected as C_5_H_9_^+^ at *m/z =* 69, however, this signal also can be attributed to a water–methanol cluster-ion, CH_3_OH (H_2_O)H^+^. If the ratio between *m/z* = 69 and its isotope at *m/z* = 70 indicates a ^13^C abundance close to the expected value for a 5-carbon compound (5.5 %) the signal measured at *m/z* = 69 is more likely to be derived from isoprene. Additionally, in complex gas mixtures, such as the ones derived from plant headspaces or human breath, it is common practice to proceed along these lines for compound identification (Lindinger et al., 1998; Crespo et al., 2011). Additional information about the identity of the molecule species also can be obtained from any other element with stable isotopes. Examples are nitrogen with an isotopic ^15^N/^14^N ratio of 0.366 %, hydrogen with a ^2^H/^1^H ratio of 0.015 %, and sulfur with a ^34^S/^32^S ratio of 4.21 %. However, ion-trap-based PTR-MS with the ability to perform MS/MS, TOF-based PTR-MS with high mass resolution, or coupling of GC with PTR-MS, are the preferred options for the unambiguous identification of compounds (Joó et al., 2010).

Basically, there are two main issues associated with the identification of compounds. First of all, the signal of the parent and fragment ions (isobaric ions) from different compounds can be superimposed on one *m/z* in the spectra without the possibility of discrimination. This complicates straightforward identification of VOCs in complex mixtures. Moreover, compounds with different structures but the same molecular mass appear at the same *m/z* signal and cannot be distinguished with a quadrupole mass filter (e.g., different monoterpenoids). To overcome these limitations, several new technologies have been developed. Combining PTR-MS with a GC, in which the VOCs are first separated by their retention time in the GC and then detected one by one by PTR-MS, avoids the overlap of different compounds and fragments (Warneke et al., 2003). Proton-transfer-reaction ion-trap mass spectrometry (PIT-MS) is another promising development to differentiate between different compounds with similar masses (Steeghs et al., 2004). This technique has characteristics similar to those of the PTR-MS, except that an ion trap is used, instead of a quadrupole as a mass analyzer. In PIT-MS, collision-induced dissociation (CID) is performed inside the ion trap, allowing different compounds with an identical mass to be differentiated by their fragmentation pattern (MS/MS). This approach enables, for example, the identification of different terpenoids and their oxygenated derivatives. Very promising is the recent development of a high-resolution time-of-flight (TOF) based system, PTR-TOF-MS, which is able to distinguish between isobaric molecules and allows unambiguous identification based on exact masses (Blake et al., 2004; Ennis et al., 2005; Graus et al., 2010). In classical PTR-MS, only one type of precursor ion (H_3_O^+^) is commonly employed to ionize compounds. In addition, other ions such as NO^+^ and O_2_^+^ can be produced in the ion source with the switchable reagent ions (SRI) technology (Jordan et al., 2009). These primary ions allow compounds with proton affinities lower than that of water (e.g., halogenated hydrocarbons) to be detected and isomeric compounds to be distinguished.

## Applications of PTR-MS for Biological Research

Since its development, PTR-MS has found many applications in a wide range of fields, including medicine (Cristescu et al., 2011), environmental sciences and atmospheric chemistry (de Gouw and Warneke, 2007; Bamberger et al., 2010; Ruuskanen et al., 2011), food monitoring (Raseetha et al., 2011), monitoring for safety and security at the workspace (Hansel et al., 1995), VOC emissions from plants during various abiotic stress conditions (Gray et al., 2010; Ruuskanen et al., 2011), and, most importantly in the context of this review, in understanding the chemistry of plant-herbivore interactions (Schaub et al., 2010; Brilli et al., 2011).

The VOC emissions resulting from plant-herbivore interactions are highly complex and dynamic. PTR-MS offers the opportunity to follow these processes in real-time. It has proven extremely difficult to use conventional sampling techniques and GC platforms to follow the fast conversion processes taking place in the lipoxygenase pathway (LOX) immediately after leaf wounding. With PTR-MS, this process has been studied at a high time-resolution, which yielded new insight into the regulation of this pathway (Fall et al., 1999; D’Auria et al., 2007). PTR-TOF-MS enabled the timing of the enzymatic conversions in the LOX pathway to be elucidated in mechanically wounded *Dactlylis glomerata* plants (Brilli et al., 2011). The conversion processes were analyzed from the initial membrane breakdown, resulting in fast emissions of C6 aldehydes, until the somewhat slower conversion of the intermediate C6 alcohols into hexyl and hexenyl acetates. Mobile PTR-MS equipment also has been used to investigate the timing of herbivore-induced green leaf volatiles, monoterpenoids, and sesquiterpenoids in poplar trees in the field (Schaub et al., 2010) and to monitor VOC emissions from complex vegetations such as grasslands or forest canopies (Davison et al., 2008; Bamberger et al., 2010; Ruuskanen et al., 2011).

However, the examples above all relate to plant volatiles induced above ground. To our knowledge, only one study has investigated root VOCs by means of PTR-MS. This study analyzed VOC emissions of *in vitro* cultured *Arabidopsis* roots after infection with a pathogen, *Pseudomonas syringae*, and the aphid *Diuraphis noxia*. The infections induced several simple metabolites, such as acetic acid, acetone, and ethanol, and a single monoterpenoid, namely 1,8-cineole (Steeghs et al., 2004). Interestingly, GLVs were not found to be released by damaged *Arabidopsis* roots. As the roots were grown *in vitro,* however, the question remains how representative the herbivore-induced responses observed in this experiment are for plants that are growing in the soil.

**Fig. 2:**
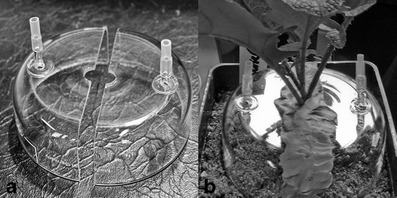
Cuvette used for dynamic headspace collections from plant roots. **a** the cuvette which consists of two parts with an air in- and outlet, respectively. **b** cuvette fitted together and tightened with a rubber-based sealant

**Fig. 3:**
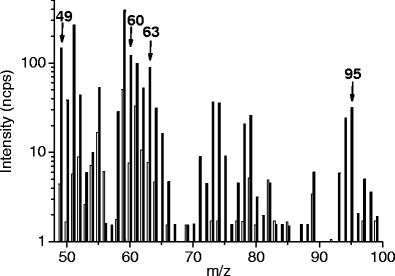
Identification of enhanced signals at masses correlated to volatile organic compounds (VOCs) from *Brassica rapa spp. rapa Nancy* by PTR-MS (*scan mode*) after root herbivory by *Delia radicum* (*black bars*) vs. control plants (*white bars*). [ncps]—normalized counts per second, *m/z* = 49—methanethiol, *m/z* = 60—related to glucosinolate breakdown products, *m/z* = 63—dimethylsulfide (DMS), *m/z* = 95—dimethyldisulfide (DMDS)

Therefore, we present two examples of preliminary PTR-MS results on herbivore-induced root responses in *Brassica* species obtained with a custom-made PTR-MS described in detail in Boamfa et al. (2004). We monitored VOCs emanating from roots of potted turnip plants (*Brassica rapa* subsp. *rapa* var. Nancy) during infestation with a belowground herbivore, the larvae of the cabbage root fly (*Delia radicum*). The root headspace of infested and non-infested plants was sampled from a cuvette fitted around the base of the stem (Fig. 2). The two parts of the cuvette were sealed together with Terostat IX (Henkel, UK), a solvent–free, rubber-based sealant (Crespo, 2012) to prevent ambient air from entering. During measurements, an excess flow of hydrocarbon-free air into the cuvette was maintained, similar to a typical dynamic headspace collection setup (Tholl et al., 2006). The resulting mass scan (Fig. 3) shows that the intensities of several molecular masses are enhanced in root fly infested *B. rapa* roots, the identities of which were confirmed by Crespo (2012) by additional GC-MS analysis and PTR-MS measurements of authentic standards. The induced intensities were detected in several structurally related sulfides, some of which have been shown to be induced in more than one *Brassica* species after root fly feeding, and which are exploited as cues by parasitoids and predators (Ferry et al., 2007; Soler et al., 2007). The mass-charge ratios representing dimethyl disulfide (DMDS; *m/z* = 63) and dimethyltrisulfide (DMTS; *m/z* = 95) displayed considerable increases in emission rates due to herbivore feeding. In addition, we also found that the biosynthetically related compound methanethiol (*m/z* = 49) was emitted at higher rates when root fly larvae were feeding. Previous GC analyses have not detected methanethiol, which might originate from the compound selectivity of the adsorbents that have been used for collection. Interestingly, we also found a considerable increase in *m/z* = 60, which is related to glucosinolate breakdown products (Crespo, 2012). This is a typical characteristic of members of the *Brassicaceae* after tissue damage. After disruption of the cells, a separately stored enzyme (myrosinase) converts the glucosinolates that are stored in the vacuoles into toxic and volatile products, such as isothiocyanates and nitriles (Hopkins et al., 2009). After activation of this two-component defense mechanism, the volatile conversion products are detected in the headspace of damaged plants (Soler et al., 2007; Pierre et al., 2011b). Our preliminary experiment shows that glucosinolate conversion products also emanate from roots, damaged by soil herbivores.

**Fig. 4:**
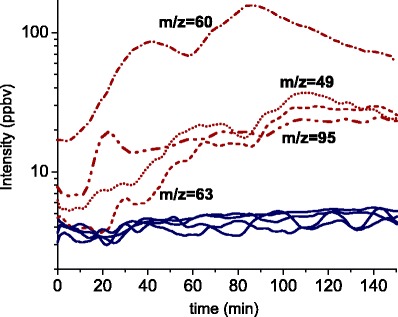
Temporal dynamics of volatile organic compound emission from *Brassica juncea* after root herbivory by *Delia radicum* (*broken lines*) and a control without damage (*continuous lines*) by PTR-MS (SIM mode). *m/z* = 49—methanethiol, *m/z* = 60—related to glucosinolate breakdown products, *m/z* = 63—dimethylsulfide (DMS), *m/z* = 95—dimethyldisulfide (DMDS)

As another example, we monitored the induction of VOCs in *B. juncea* roots after infestation with *Delia radicum* in real-time and compared it to a control treatment (Fig. 4). We followed the emission of root VOCs for several hours in SIM mode, starting immediately after ten actively feeding second instar larvae were added to the roots. Based on the previous example, we chose to record specifically the masses which correlate to the three sulfides from the previous experiment and the mass 60, all of which already revealed differences between the treatments in scan mode (Fig. 3). Initially, we observed a low emission rate of only several ppbv for these compounds, which steadily increased with longer feeding times of the root flies (Fig. 4). In control plants, the VOC emissions remained at a very low level, which allowed a clear distinction between control and infested plants within a few hours after infestation. We suggest that further development of PTR-MS methods and sampling set-ups might provide us with the tools to correlate the intensities of the VOC emissions directly to the amount of herbivore damage in a quantitative manner. Possibly, the PTR-MS emission patterns can be used to assess the infestation level of root herbivores or to assess the time point when they stop feeding or start pupating by exploiting certain VOC related masses as non-invasive markers.

## Conclusions

As outlined, PTR-MS has in the recent years opened an avenue for new insight into fast changing, highly dynamic processes involved in plant VOC emissions caused by plant-environment interactions. Here, we show that, due to its sensitivity and the ability to record real-time responses, PTR-MS is an excellent technique to non-invasively trace the feeding activities of cryptically feeding root herbivores by measuring VOC emissions from the root headspace. Certainly, PTR-MS also has its practical and technical limitations. Besides difficulties in linking masses without doubt to compounds, many quadrupole-based systems lack sensitivity in the higher mass range (above 120 amu), which is relevant for plant-herbivore interactions, as many biologically important compounds, such as several isothiocyanates or generally hemiterpenoids and sesquiterpenoids are difficult to detect. These shortcomings can be overcome partially by combining on-line sampling with PTR-MS and off-line GC-MS methods, or by use of high sensitivity PTR-MS instruments with mass analyzers, such as distinctive quadrupoles, triple quadrupole technology, ion trap, or time-of-flight, which can provide sensitivity also in the higher mass range (Tani et al., 2003; Kim et al., 2009). We expect that the innovative and fast-evolving field of MS technologies will result in further improvements regarding sensitivity and mass resolution. Consequently, PTR-MS-based technologies may soon approach detection limits even closer to the sensitivity of insect antennae. A recently developed sensor, for example, which uses antennae of Colorado potato beetles (*Leptinotarsa decemlineata*) or of jewel beetles (*Phaenops cyanea*), demonstrates that insect antennae are capable of detecting, for instance, the GLV (*Z*)-3-hexen-1-ol at around 1 ppmv and 1 pptv, for the two species, respectively. Compared to that, the detection limits in PTR-MS already are in a similar range of several parts per trillion volume, depending on the properties of the instrumentation.

Finally, time-resolved and sensitive on-line sampling of root-induced volatiles with PTR-MS will certainly contribute to our understanding of the role of VOCs in belowground multi-trophic interactions. In particular, linking real-time responses in the emission of VOCs to immediate behavioral responses of herbivores, and to the higher trophic levels of parasitoids and predators will unravel further details of the VOC ‘language’ among plants and between plants and insects. This may be achieved by ‘sniffing out’ the VOCs in parallel with olfactometer assays, a prime example of which is the development of a six-arm olfactometer, simultaneously equipped with a VOC sampling unit (Turlings et al., 2004). In a similar way, coupling PTR-MS analyses with microbial bioassays may help to disentangle the impact of belowground plant VOCs on other soil organisms, such as pathogens and microbes (Effmert et al., this issue). In addition to that, new approaches in multivariate statistical analyses will facilitate discrimination of biologically meaningful information from noise contained in these increasingly complex mixtures (van Dam and Poppy, 2006; Jansen et al., 2010) with steadily increasing numbers of compounds due to the rising sensitivity of instrumentation. Last but not least, improving our methods for non-destructive plant VOC sampling from soil environments will complement the knowledge we have gained already from aboveground plant organs, with further discrepancies and similarities being discovered between the two compartments of plants living apart together. With a more complete perspective on plant VOCs and their biological roles, we might later be able to complement our current perceptions of plant defenses, adapted to a perspective of the whole plant.

## Footnotes

1 The formula assumes an ideal gas at T = 25°C and P = 1 atm. For more precise conversions, parameters such as the true temperature during sampling, need to be accounted for. The molar volume of an ideal gas at these conditions is 24.5 l·mol^-1^. **M**_**r**_—relative molecular mass, **DW**—dry weight, **m**_**plant**_—total plant dry weight of sampled plant part, **V**_**air**_—total volume of air sampled.
